# Extracellular vesicles in oral squamous carcinoma carry oncogenic miRNA profile and reprogram monocytes via NF-κB pathway

**DOI:** 10.18632/oncotarget.26208

**Published:** 2018-10-05

**Authors:** Fatemeh Momen-Heravi, Shashi Bala

**Affiliations:** ^1^ Division of Periodontics, Section of Oral and Diagnostic Sciences, College of Dental Medicine, Columbia University, New York, NY, USA; ^2^ Department of Medicine, University of Massachusetts Medical School, Worcester, MA, USA

**Keywords:** extracellular vesicles, miRNAs, NF-κB, head and neck cancer, exosomes

## Abstract

Extracellular vesicles (EVs) are carriers of different biomacromolecules that participate in cellular signaling and disease pathogenesis. Although it has been shown that EVs can play an active role in cellular communication and different stages of cancer progression, the role of EVs in oral squamous cell carcinoma (OSCC) cancer pathogenesis, especially in the crosstalk of cancer cells with immune cells is unknown. Here, we present a detailed analysis of findings regarding the profile of EVs in OSCC and the role of EVs and associated miRNAs in the crosstalk of malignant cells with monocytes. We demonstrate that EVs are detectable in significantly higher quantities in the plasma of patients with OSCC. Oncogenic miRNAs (such as miR-21, miR-27) were detectable in high quantities in the circulating EVs and plasma of patients with OSCC. EVs isolated from the circulation of OSCC patients and OSCC cell lines showed comparable miRNA signature, indicating the tumor origin of EVs in the circulation of patients with OSCC. Danger signals such as LPS and ethanol increased the production of EVs. EVs were taken up by monocytes after co-culture. Mechanistically, uptake of EVs derived from oral cancer cells by monocytes caused activation of the inflammatory pathway, NF-κB activation, and establishment of a pro-inflammatory and pro-tumorigenic milieu marked by increased levels of IL-6, CCL2, PEG2 and MMP9 levels. Series of experiments involving the introduction of exogenous oncogenic miR-21 mimic induced a similar pro-inflammatory and pro-tumorigenic profile in monocytes. Inhibiting miR-21 function in monocytes attenuated the pro-inflammatory phenotype of monocytes after EV challenge. These results indicate the role of EV-associated miR-21 in modulating the immune response in monocytes.

## INTRODUCTION

Oral cancer is a global health problem that demonstrates a challenge to the health care system [1]. Oral squamous cell carcinoma (OSCC) accounts for more than 90% of oral cancers [1, 2]. Several risk factors for OSCC such as smoking, alcohol consumption, smokeless tobacco use, and infection with human papillomavirus have been reported [3–5]. Despite the advances in the cancer treatment the 5-year survival of oral cancer is around 50% in various stages of the disease [6]. However, more than 60% of patients present with stage III or IV of the disease at the time of diagnosis which leads to even less survival rate. Therefore, a better understanding of the initial events in OSCC is needed to improve early detection and disease intervention.

Extracellular vesicles (EVs), membrane vesicles of cell origin, are emerging as key players in intercellular communication between cells and their microenvironment through horizontal transfer of cargo which contains proteins, DNAs, messenger RNAs, and microRNAs [7–10]. Extracellular vesicle is a collective term for exosomes and microvesicles. Exosomes are the smallest (30–100 nm) subpopulation of EVs and are originated from multivesicular bodies. Microvesicles (also called shedding vesicles, shedding microvesicles, or microparticles) are approximately 100–1000 nm in diameter and originate from the outward budding of the plasma membrane [11, 12]. EVs have been isolated from almost all cell types and different bio-fluids including blood, urine, cerebrospinal fluid, and saliva [11]. They have been implicated in critical cellular processes such as growth and development, cell-to-cell communication, immunomodulation, blood coagulation, and various stages of tumorigenesis [13–15].

Several groups have demonstrated that presence of pathological states such as oxidative stress, transformation, apoptosis, and alcohol-induced cell injury trigger cells to increase their EV release rate, simultaneously altering their composition to reflect the altered state of the cellular origin [16–18]. Upon secretion, EVs can be taken up by other cells and modulate the recipient cell function through horizontal transfer of biomacromolecules or activation of surface receptors [19, 20]. Recently, the role of EVs in various phases of cancer initiation, cancer progression, and metastasis formation was shown in different cancers such as pancreatic cancer, colon cancer, lung cancer, malignant melanoma, and breast cancer [21–25]. Studies have shown the role of EVs in oral cancer [26, 27]. However, immune response mediated by EVs originated from oral cancer cells is not known.

Evidence regarding molecular and cellular mechanisms underlying cancer development indicates that immune changes occur during cancer development and progression. Alcohol is considered one of the risk factors for oral squamous cell carcinoma (OSCC) and associated with poor prognosis which is also a modulator of innate immunity [28]. Here, we present a detailed analysis of the role of EVs and associated miRNAs in cellular crosstalk in OSCC. We demonstrate that EVs are detectable in significantly higher quantity in circulations of patients with OSCC and the majority of extracellular RNA are packed in the EVs. Oncogenic miRNAs were detectable in high quantity in the circulating EVs and plasma of patients with OSCC. We also performed a series of experiments to characterize the EVs shed by oral cancer cells in resting state and in response to the alcohol challenge. We found that alcohol challenge increases EV production in a dose-dependent manner and subset of oncogenic miRNAs are specifically enriched in the EVs released form the cancer cells. Mechanistically, uptake of EVs derived from oral cancer cells by monocytes causes activation of the NF-κB pathway and establishment of the pro-inflammatory milieu. These findings suggest that EV-associated miRNAs may be a marker, as well as a functional component, of cancer cells and immune cells interactions. In summary, our study describes a previously unknown pro-inflammatory circuit through which OSCC-derived EVs can induce the formation of pro-inflammatory phenotype in monocytes.

## RESULTS

### Extracellular vesicles are increased in patients with OSCC and carry oncogenic miRNAs

**Figure 1:**
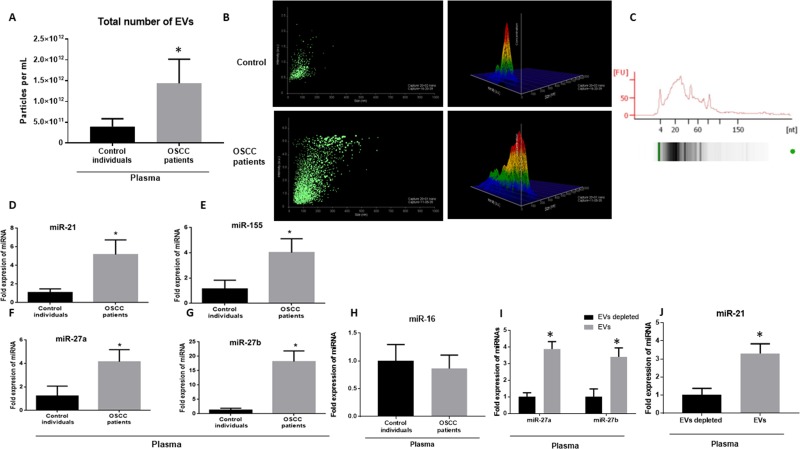
Number of EVs and oncogenic miRNAs are increased in the plasma of patients with OSCC (**A**) Total number of EVs in the plasma of patients with OSCC and matched controls were measured by a Nanoparticle Tracking analysis (Nanosight) (*n* = 34). Each measurement was done in triplicate. (**B**) The average size of exosomes was measured by Nanoparticle Tracking analysis (NTA) in control and OSCC patients. (**C**) The graph is representative of small RNA of EVs isolated from plasma of patients with OSCC as analyzed by BioAnalyzer. (**D**–**H**) Signature of plasma miRNAs in patients with OSCC and controls was assessed with a TaqMan miRNA qPCR assay for quantification of miR-21, miR-155, miR-27a, miR-27b, miR-16. miR-Cel-39 was used as exogenous normalizer. (**I**, **J**) Expression levels of miR-27a, miR-27b, and miR-21 was identified in EV fraction and non-EV fraction of plasma starting from same amount of plasma (100 μl) with a TaqMan miRNA qPCR assay (*n* = 10 control, 10 OSCC patients). After isolation of EVs RNase A treatment at final concentration of 5 μg/mL for 30 min was done. (^*^indicates *p* < 0.05 *versus* control).

We examined the number and composition of EVs in patients with OSCC and gender-matched controls. The baseline characteristic of human subjects is presented in Supplementary Table 1. We enumerated extracellular vesicles in the plasma of patients with OSCC, using a nanoparticle tracking analysis (NTA) system. We found an increased number of EVs in plasma of patients with OSCC compared to the controls (up to 3.2 times) (*p* < 0.001) (Figure 1A). The size of extracellular vesicles was significantly larger in patients with OSCC compared to healthy controls with the average of 389 nm in OSCC and 178 nm in controls (*p* < 0.05). The data indicate that the extracellular vesicles were mostly in the range of a larger group of extracellular vesicles called microvesicles or microparticles (Figure 1B). Circulating EVs carried small RNA cargo as represented in bioanalyzer results obtained by small RNA chip (Figure 1C). miRNA profile of circulating miRNA revealed increased levels of oncogenic-related miRNAs including miR-21, miR-27a, miR-27b, and miR-155 in the plasma of patients of patients with OSCC compared to the controls with the average fold changes of 5.16, 4.1, 12.80, and 4.2 respectively (*p* < 0.05) (Figure 1D–1G). In contrast, miR-16 showed no change in plasma of patients with OSCC compared to the controls (*p* > 0.05) (Figure 1H). Profiling of EVs isolated from plasma of patients with OSCC showed that miR-21, miR-27b, and miR-27a were specifically enriched in EVs compared to the non-EV fraction (Figure 1I, 1J).

### Oral cancer cell line produces an increasing number of extracellular vesicles after stimulation with danger signals

Since we found an increased number of EVs in patients with OSCC, we postulated that the origin of the EVs were OSCC cells. We further study the CAL27 cell line (ATCC^®^ CRL2095™), a human oral squamous cell carcinoma cell line, to characterize the content and function of EVs. We found that OSCC cells shed a considerable amount of EVs and those EVs were increased after stimulation with danger signals including ethanol and LPS.

**Figure 2:**
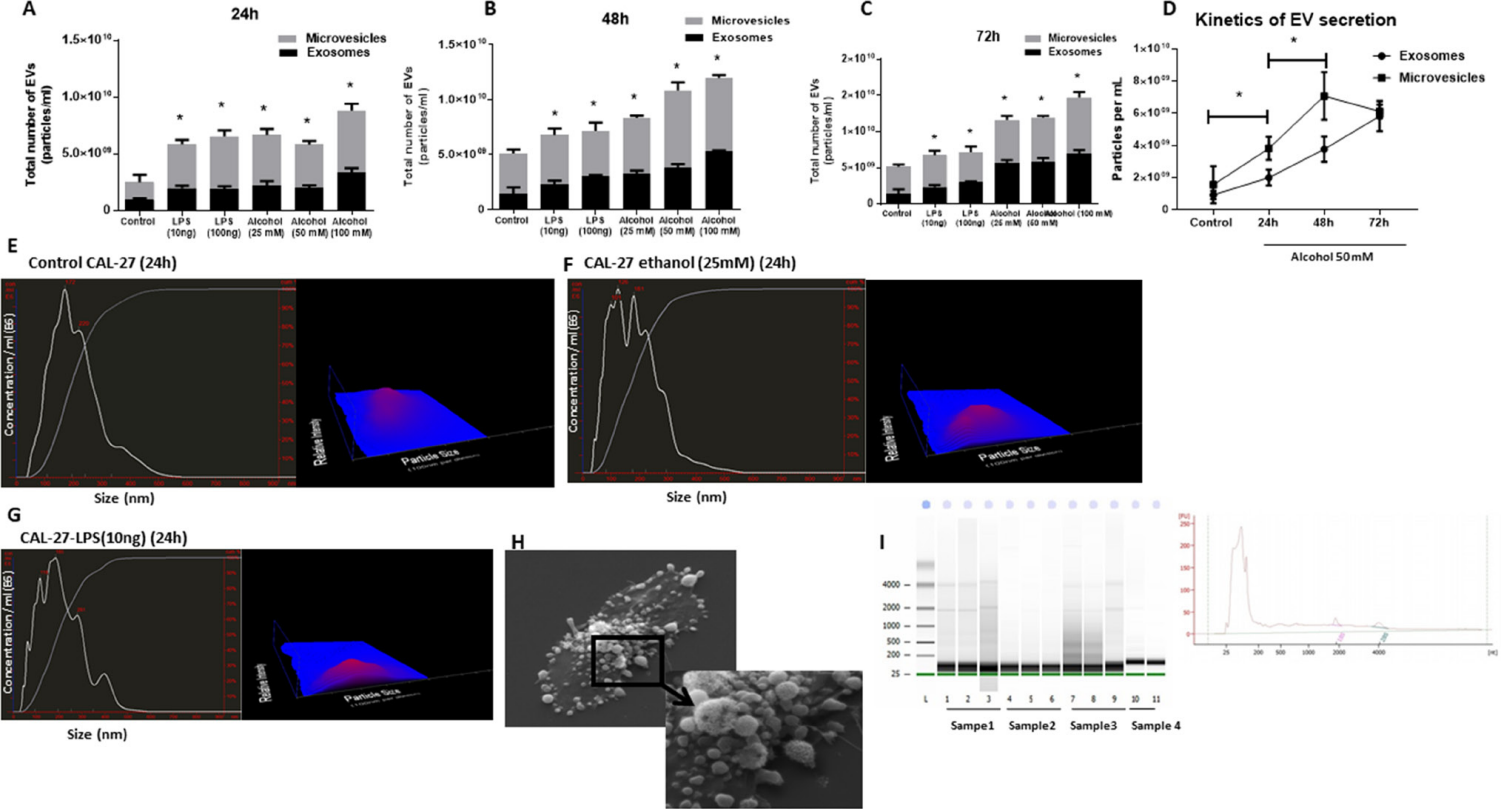
Ethanol treatment increases total number of EVs in a dose- and time-dependent manner in CAL27 cells CAL27 cells were cultured, and numbers of EVs, exosomes (size <150 nm), and MVs (size >150 nm) were quantified in cell-free supernatant by NanoSight. (**A**) CAL27 cells were exposed to various dose of ethanol (25 mM, 50 mM, 100 mM), LPS (10 ng, 100 ng) or left untreated for 24 h, and the frequency of vesicles was determined. (**B**) CAL27 cells were exposed to various dose of ethanol (25 mM, 50 mM, 100 mM), LPS (10 ng, 100 ng), or left untreated for 48 h, and the frequency of vesicles was determined. (**C**) CAL27 cells were exposed to various dose of ethanol (25 mM, 50 mM, 100 mM), LPS (10 ng, 100 ng), or left untreated for 72 h, and the frequency of vesicles was determined. (**D**) Kinetic of EV production after challenge with 50 mM ethanol was determined at each condition by enumerating the number of EVs in the cell supernatants. (**E**) Size distribution of EVs produced by CAL27 cells in 24 h in the unchallenged condition. (**F**) Size distribution of EVs produced by CAL27 cells challenged by 25 mM ethanol in 24 h. (**G**) Size distribution of EVs produced by CAL27 cells challenged by 10 ng LPS in 24 h. Nanosight device was calibrated with 100 nm polystyrene beads before each set of measurements. All measurements were done in triplicate from 6 independent samples in each group. (**H**) Scanning electron microscopy image of untreated CAL27 cells are shown (×3000 magnification). The blown-up image of the selected region (×7500 magnification) in the cancer cells shows the shedding of EVs on the surface of the cells. (**I**) EVs originated from CAL27 cells carry small noncoding RNAs in size range of miRNAs. Figure is the representation of EV-encapsulated small RNA profile obtained from 4 independent sample preparations. (^*^indicates *p* < 0.05 *versus* control).

In a series of experiments, we challenged OSCC cells with a different dose of LPS and ethanol to identify the effect of ethanol and LPS stimulation on the EV production and miRNA signature. Administration of 10 ng/ml and 100 ng/ml LPS significantly increased a total number of extracellular vesicles in CAL27 cells. Similarly, administration of 25 mM, 50 mM, 100 mM ethanol for 24 h, 48 h, and 72 h which are clinical equivalent of mild ethanol exposure, moderate ethanol exposure, and severe ethanol exposure [29], showed a significant increase in the number of extracellular vesicles (Figure 2A–2D). Interestingly, administration of both LPS and ethanol challenges induced more increase in the larger subset of extracellular vesicles (microvesicles) compared to the smaller subpopulation (exosomes) and mean diameter of extracellular vesicles was increased significantly after 24 h challenge with 25 mM ethanol and 10 ng/ml LPS (Figure 2E–2G). EVs released after alcohol challenge (25 mM) showed the increased average size of 348 nm compared to the control cells with an average size of 162nm. Characterization of extracellular vesicles from CAL 27 cells with western blot revealed the presence of EV marker CD63 (Supplementary Figure 1). RNA encapsulated in EVs was protected against RNases in both EVs isolated from plasma and culture media of CAL27 cells (Supplementary Figure 2). CAL27 cells produce extracellular vesicles as shown in the scanning electron microscopy image (left image). Higher magnification reveals EVs ranging from 50–500 nm in diameter (Figure 2H). We then conducted bioanalyzer capillary electrophoresis on all EV-associated RNA to identify their size distributions. Small species ranging in size from <150 nt were most prevalent in all examined electrophoretic profiles, although signatures of longer transcripts were also present (Figure 2I).

### EVs derived from oral squamous cell carcinoma cells are enriched in oncogenic miRNAs

**Figure 3:**
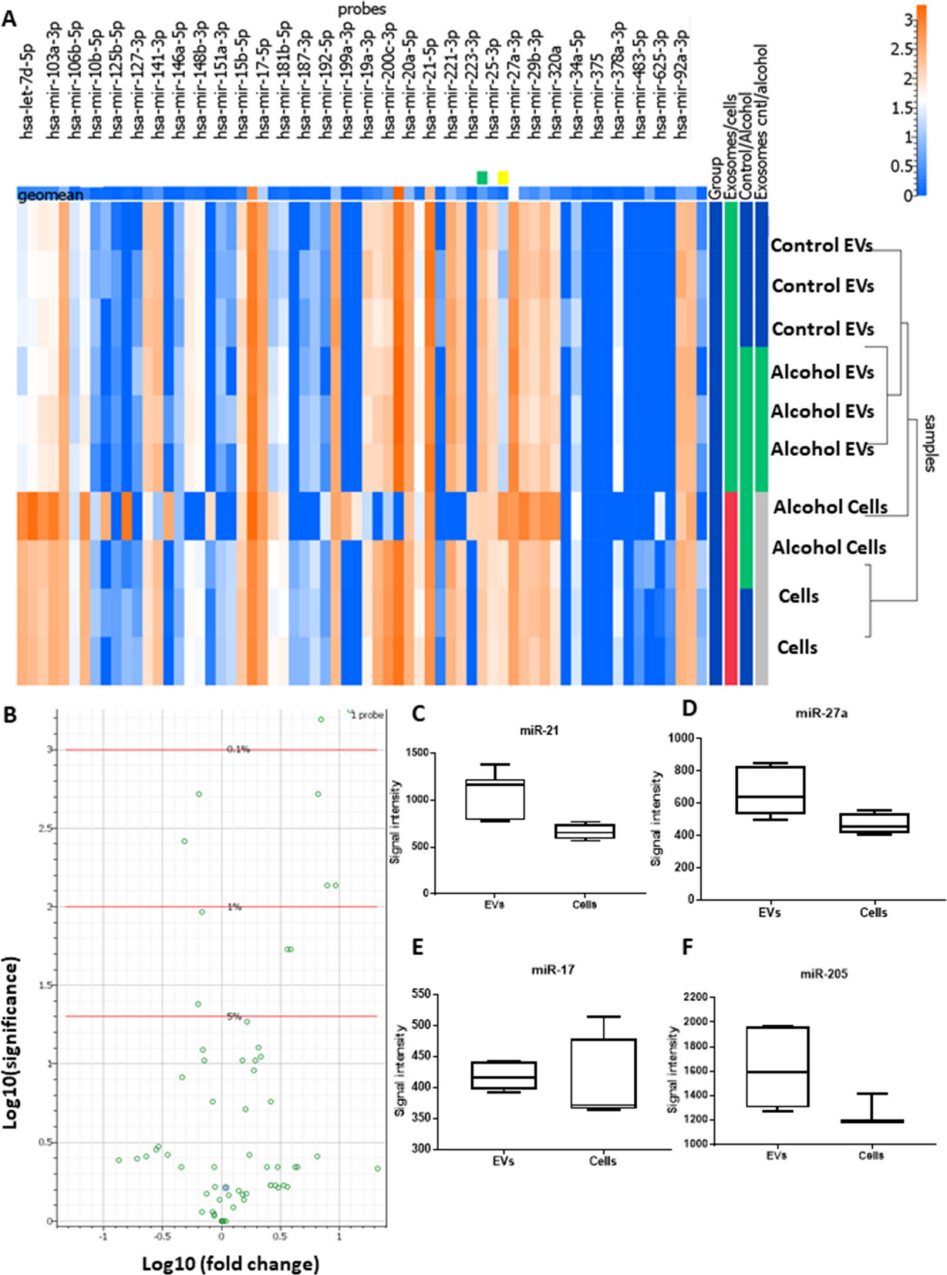
Characterization of miRNA cargos of EVs secreted by OSCC cells (CAL27) (**A**) Heat map of differentially expressed EV-associated miRNAs in oral squamous cell carcinoma cell lines (CAL27) using Firefly BioWorks miRNA assay normalized to the geometric mean of most stable miRNAs. (**B**) Volcano plot of significant relative to the fold change expression of miRNAs for cell versus EVs. (**C–F**) Normalized signal intensity of miR-21, miR-27a, miR-205, and miR-17 in CAL27 cell and EVs (^*^indicates *p* < 0.05 *versus* control).

Next, we hypothesized that EVs derived from oral squamous cell carcinoma contain oncogenic miRNAs. To identify deregulated miRNAs in cell line and EVs in the absence and presence of ethanol, we challenged cells with ethanol 25 mM for 24 h and miRNA analysis was carried out using the Firefly miRNA multiplex assay for oncology panel. Interestingly, the miRNA profile of EVs derived from ethanol challenged cells was mostly similar to the EVs isolated from non-challenged cells with the exception of 5 miRNAs: miR-378a-3p, miR-130a-3p, miR-181a-5p, miR -205-5p, and miR-122-5p (Table 1). This data indicates that the effect of ethanol on the cancer cells is most effective on the magnitude of production of EVs and a subset of miRNAs packaging. miRNAs were selectively enriched into the EVs, and the profile of miRNA was different in EVs and the cells (Figure 3A, 3B). The packaging of cellular miRNAs into the EVs was specific, and some of the top miRNAs which differentially expressed between cells and EVs were: miR-7d-5p, miR-130a-3p, miR-181a-5p, miR-107, miR-21-5p, miR-103a-3p, miR-22-3p, miR-20a-5p (Table 1).

**Table 1:** List of differentially expressed miRNAs in OSCC cells and EVs

	Fold	adjusted *p*-value	raw *p*-value
Alcohol versus control			
hsa-mir-146a-5p	1.47	0.525	0.011
hsa-mir-27a-3p	1.32	0.965	0.050
Control EVs versus Alcohol EVs			
hsa-mir-378a-3p	1.27	0.463	0.009
hsa-mir-130a-3p	0.78	0.758	0.021
hsa-mir-181a-5p	1.81	0.928	0.039
hsa-mir-205-5p	1.34	0.929	0.039
hsa-mir-122-5p	0.20	0.958	0.047
hsa-mir-146a-5p	1.60	0.964	0.049
hsa-mir-181b-5p5p	1.95	0.964	0.049
EVs versus cells			
hsa-mir-378a-3p	1.27	0.463	0.009
hsa-mir-130a-3p	0.78	0.758	0.021
hsa-mir-181a-5p	1.81	0.928	0.039
hsa-let-7d-5p	13.33	0.001	0.000
hsa-mir-107	7.53	0.002	0.000
hsa-mir-200b-3p	2.96	0.010	0.000
hsa-mir-103a-3p	7.08	0.010	0.000
hsa-mir-130a-3p	0.52	0.013	0.000
hsa-mir-22-3p	0.69	0.014	0.000
hsa-mir-20a-5p	0.74	0.042	0.001
hsa-mir-148a-3p	0.76	0.043	0.001
hsa-mir-26a-5p	10.05	0.054	0.001
hsa-let-7g-5p	8.58	0.057	0.001
hsa-mir-148b-3p	0.75	0.100	0.002
hsa-mir-15b-5p	1.78	0.150	0.002
hsa-let-7i-5p	4.14	0.171	0.003
hsa-mir-181a-5p	3.90	0.173	0.003
hsa-mir-18a-5p	1.90	0.253	0.004
hsa-mir-106b-5p	1.62	0.337	0.006
hsa-mir-181b-5p	2.24	0.515	0.011
hsa-mir-155-5p	4.03	0.519	0.011
hsa-mir-10b-5p	2.34	0.643	0.015
hsa-mir-34a-5p	2.08	0.728	0.020
hsa-mir-21-5p	0.68	0.796	0.024
hsa-mir-29b-3p	2.03	0.825	0.026
hsa-mir-151a-3p	1.65	0.878	0.031
hsa-mir-222-3p	0.82	0.939	0.041
hsa-mir-19a-3p	0.50	0.943	0.042
hsa-mir-192-5p	2.84	0.963	0.049

Interestingly, miR-21, miR-27a, miR-205, and miR-17a showed very high signals in both cells and EVs indicating an abundance of these miRNAs in both cancer cells and released EVs (Figure 3C–3F).

### EVs miRNA signature targets multiple cancer and immune system associated pathways

**Figure 4:**
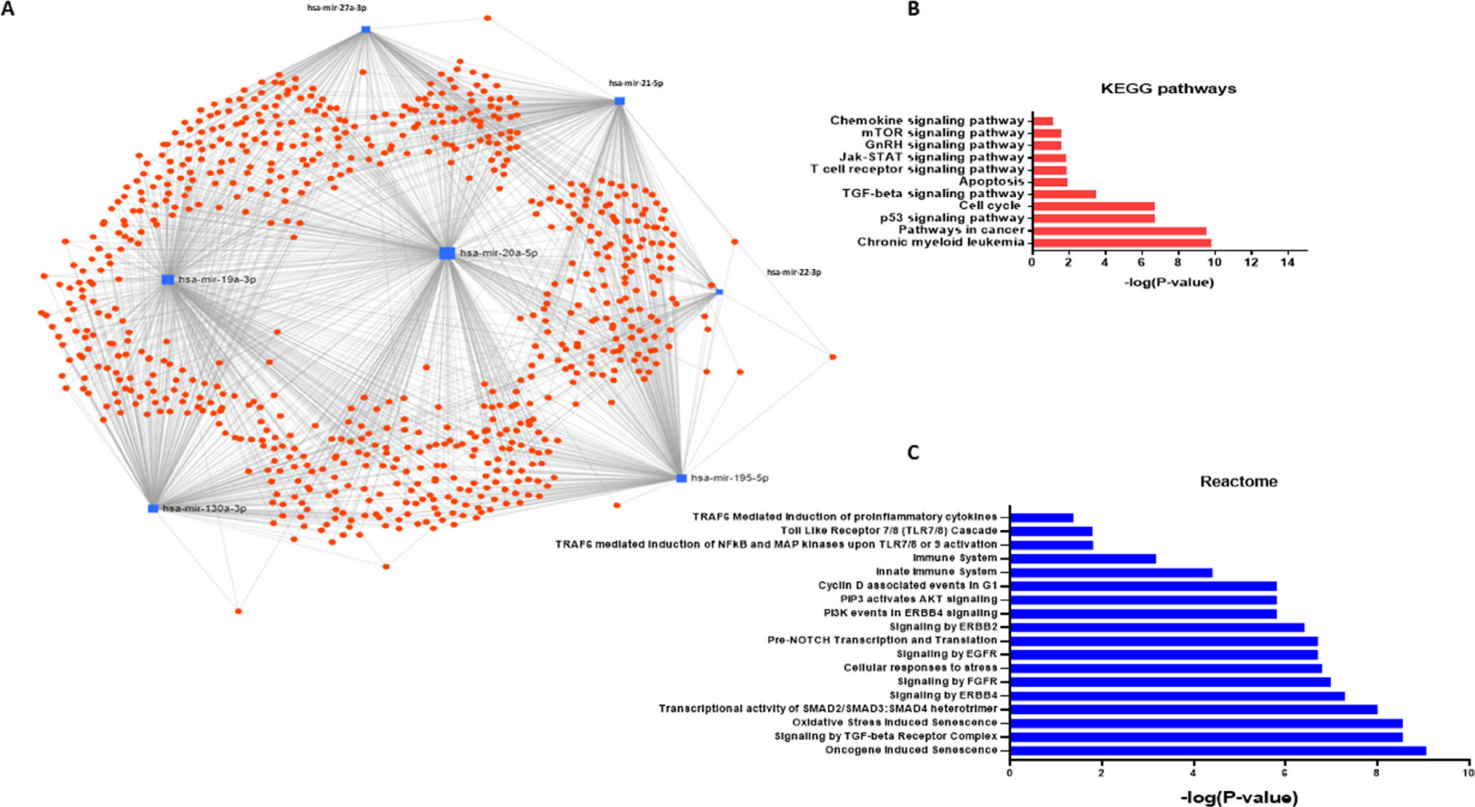
Network analysis and gene set enrichment analysis of miRNAs enriched in OSCC derived EVs (**A**) Target interaction network for miRNA enriched in the OSCC EVs. Schematic was obtained from miRNet.ca. (**B**) Selected KEGG pathway analysis significant results combining gene targets of selectively enriched miRNAs in OSCC EVs were shown. (**C**) Selected Reactome pathway analysis significant results combining gene targets of selectively enriched miRNAs in OSCC EVs were shown.

Massive miRNA-gene interaction is present in cellular pathways. For example, KEGG pathway enrichment analysis of miRNA targets has revealed tens of thousands of miRNA-gene interactions [30]. Specifically, miR-21 shows the highest regulatory activity of biological pathways by targeting over 1400 genes. miRNAs which showed significant enrichment in EVs (miR-22-3p, miR-130a-3p, miR-20a-5p, miR-21-5p, miR-195-5p, miR27a-3p, miR-19a-3p) were entered to the network-based analysis (Figure 4A). We identified 40 KEGG pathways potentially regulated by combined miRNA signature. Significant enriched pathways were “Pathways in cancer”, “P53 signaling pathway”, “mTOR signaling pathway”, “Jak-STAT signaling pathways”, “Cell cycle”, and “Chemokine Signaling Pathway”. We identified 132 Reactome pathways as potential pathways regulated by combined miRNA signature including “Oncogene-induced senescence”, “Signaling by TGF-beta receptor complex”, “Cellular response to stress”, “Toll-like receptor 7/8 cascade”, “Immune system,” and “Innate immune system.” The gene set enrichment analysis results showed this miRNA network regulates multiple pathways involved in cancer and innate immune function. Top significant pathways are shown in Figure 4B and 4C. The complete set of pathways obtained from gene set enrichment analysis are included as Supplementary Table 2 and Supplementary Table 3. Analysis of miRNA-small molecule interactions revealed that the EV miRNA signature interacts with various drugs which routinely used in cancer therapy including 5-aza-2′-deoxycytidine, 5-fluorouracil, Trichostatin A, among others (Supplementary Figure 3). These *in silico* analyses revealed the possible role of this miRNA network in cancer (p53/TGFβ/SMAD/apoptosis pathways) and immune function. In accordance with *in silico* analyses, miR-21 is shown to modulate various genes involved in p53/TGFβ/apoptotic/SMAD pathways [31] and in immunity [32]. Consistent with patient data, miR-21 showed the highest uniform signal intensity across different groups in both cells and EVs. This data confirmed by qPCR and consistently miR-21 showed lower Ct values in both CAL27 cells, and EVs derived from those cells (Ct value: 24 in 10 ng cDNA). miR-21 is an oncogenic miRNA which plays a key role in resisting programmed cell death, targeting numerous tumor suppressor genes associated with proliferation, apoptosis, and invasion [33, 34]. Overexpression and role of miR-21 as one of the essential oncomiRs in head and neck cancer has been underlined [35–38]. We hypothesized that EVs released by OSCC and increased production of EVs due to the ethanol challenge plays a role in tumor microenvironment modulation and increased miR-21 has a role in modulating monocyte functions.

### EVs derived from cancer cells are taken up by monocytes and activate NF-κB pathway

**Figure 5:**
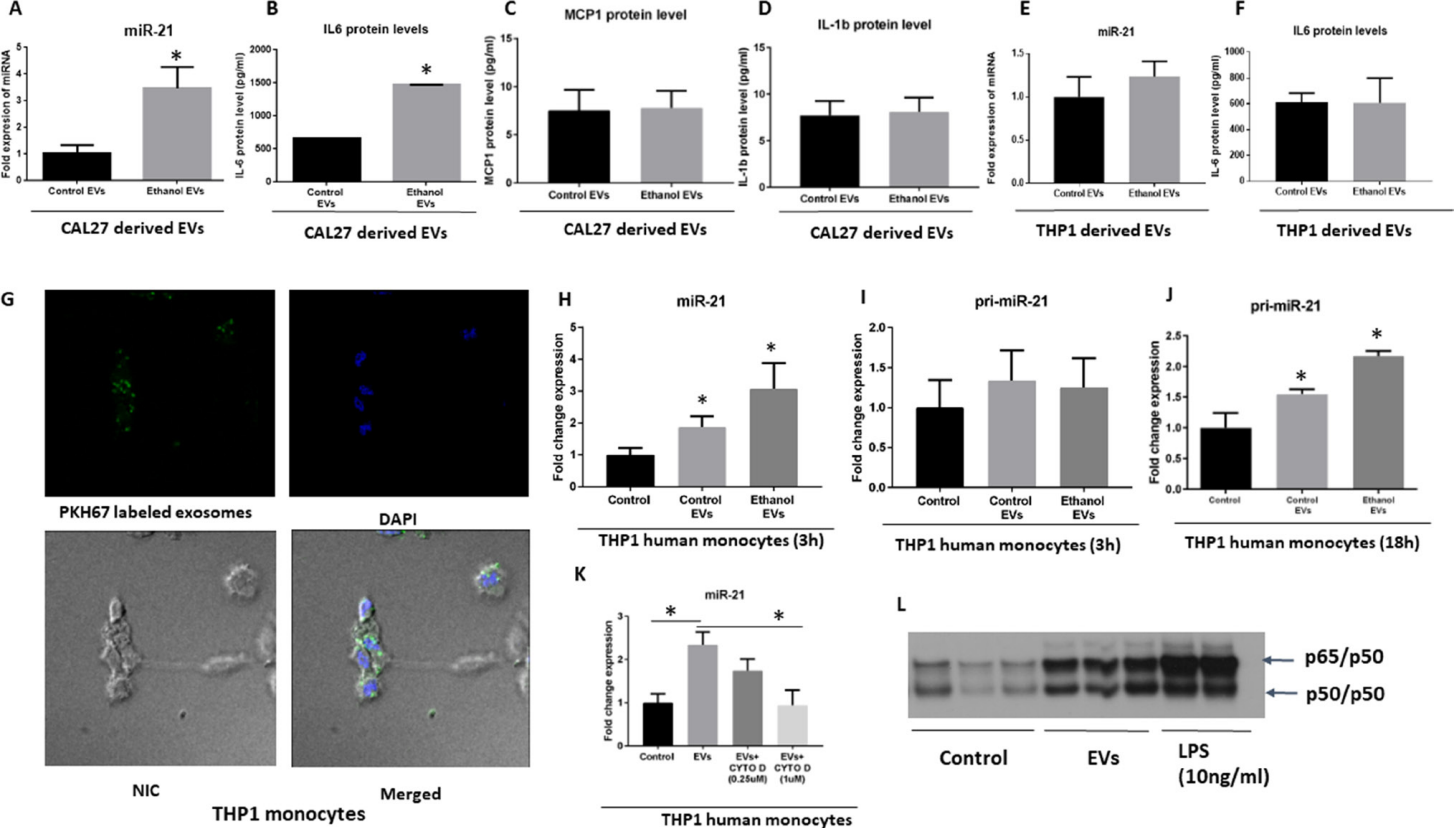
Uptake and biological activity OSCC derived EVs in human THP1 monocytes (**A**–**D**) Levels of miR-21 and IL-6 protein, MCP1 protein and IL-1β protein levels were quantified by a TaqMan Assay (miR-21) and ELISA assays respectively in EVs derived from CAL27. The quantitative results represent three independent experiments. (**E**, **F**) Levels of miR-21 and IL-6 protein were quantified by a TaqMan Assay and ELISA assays respectively, in EVs derived from THP1 cells. (**G**) EVs derived from CAL27 cells were labeled with green fluorescent dye (PKH67) and co-cultured with THP1 cells (2 h). DAPI was used to stain the nuclei. Nomarski Interference Contrast (NIC) was used for identifying the cytoplasm. EVs were taken up by the THP1 cells evidenced by the presence of green fluorescently labeled exosomes in the cytoplasm of the THP1 monocytes in the merged image. (**H**–**J**) Levels of miR**-**21 and pri-miR-21 were quantified by a TaqMan miRNA Assay and TaqMan Pri-miRNA Assay respectively at the indicated time points. (**K**) Level of miR-21 uptake was determined by qPCR after EV uptake in cells treated with varying concentrations of Cytochalasin D (CYTO D). (**L**) EVs were isolated from CAL27 cells from three independent experiments and were cocultured with human THP1 monocytes for 6 h. THP1 cells were treated or not with 10 ng/ml LPS for 6 h, and 10 μg of whole cell lysate were subjected to EMSA to evaluate NF-κB activation. (^*^indicates *p* < 0.05 *versus* control).

We further characterized the content of EVs derived from CAL27 cells with and without ethanol stimulation. To mimic the biological concentration and phenomena, we normalized the EVs RNA based on the number of parental cells. This normalization enabled us to compare more relevant biological phenomenon after ethanol challenge which accompanied by an increase in the number of EVs. EVs derived from CAL27 cells at normal condition, and under ethanol challenge contained the high signal of miR-21 (24 Ct value for normal condition and 22 Ct value after ethanol challenge) (Figure 5A). Those EVs were also highest in IL-6 protein content but no change in the levels of MCP1 and IL-1b protein content (Figure 5B–5D). To check whether the changes in the miR-21 cargo after ethanol challenge is specific to EVs derived from CAL27 cells, we used THP1 cells as a control cell line. Level of miR-21 and IL-6 were not significantly altered in THP1 cells derived EVs after ethanol challenge (Figure 5E, 5F). These findings indicate selective increase and sorting of cargos into the EVs. Further, EVs derived from CAL27 cells were labeled with PKH67 green fluorescent and were taken up by THP1 monocytes after 2 h co-culture (Figure 5G). miR-21 was increased in the THP1 monocytes after 3 h co-culture of EVs and THP1 cells (Figure 5H). Both control EVs and ethanol-induced EVs transferred miR-21 to THP1 human monocytes, with an increase in miR-21-fold expression in the THP1 monocytes which was expected since more EVs was present in the supernatant of alcohol-treated cells with the same number of parental cells (Figure 5H). We ruled out the endogenous source of miR-21 by performing primary transcript containing miR-21 (pri-miRNA-21) qPCR in the cells. No change in pri-miRNA-21 was found after 3 h of co-culture of EVs derived from OSCC in the monocytes (Figure 5I). Interestingly, pri-miRNA-21 was significantly increased 18 h after co-culture of EVs derived from OSCC in the monocytes, indicating later induction of miR-21 after internalization of EVs and *de novo* synthesis of miR-21 at later time points (Figure 5J). To further confirm that the uptake of EVs was an active process and leads to increase in miR-21 was related to the endocytosis pathways, we used Cytochalasin D. Cytochalasin D is a metabolite known to depolymerize the actin filament network resulting in inhibition of endocytic pathways and EVs uptake in a dose-dependent manner [39, 40]. Cytochalasin D treatment leads to a dose-dependent decrease in miR-21 level (Figure 5K), indicating that the uptake of EVs containing miR-21 is an active process and involves endocytosis. To determine the function of EVs in recipient THP1 monocytes we performed an electrophoretic mobility shift assay (EMSA) specific to NF-κB consensus sequences. The results showed activation of NF-κB (p65/50 heterodimers) in THP1 monocytes, 4 hours after treatment with EVs derived from CAL27 cells (Figure 5L). Consistent with other studies, LPS treatment resulted in NF-κB activation in our system (Figure 5L). These results suggest a biological role of EVs in recipient THP1 monocytes.

### EVs derived from cancer cells reprogram monocytes via NF-κB pathway

**Figure 6:**
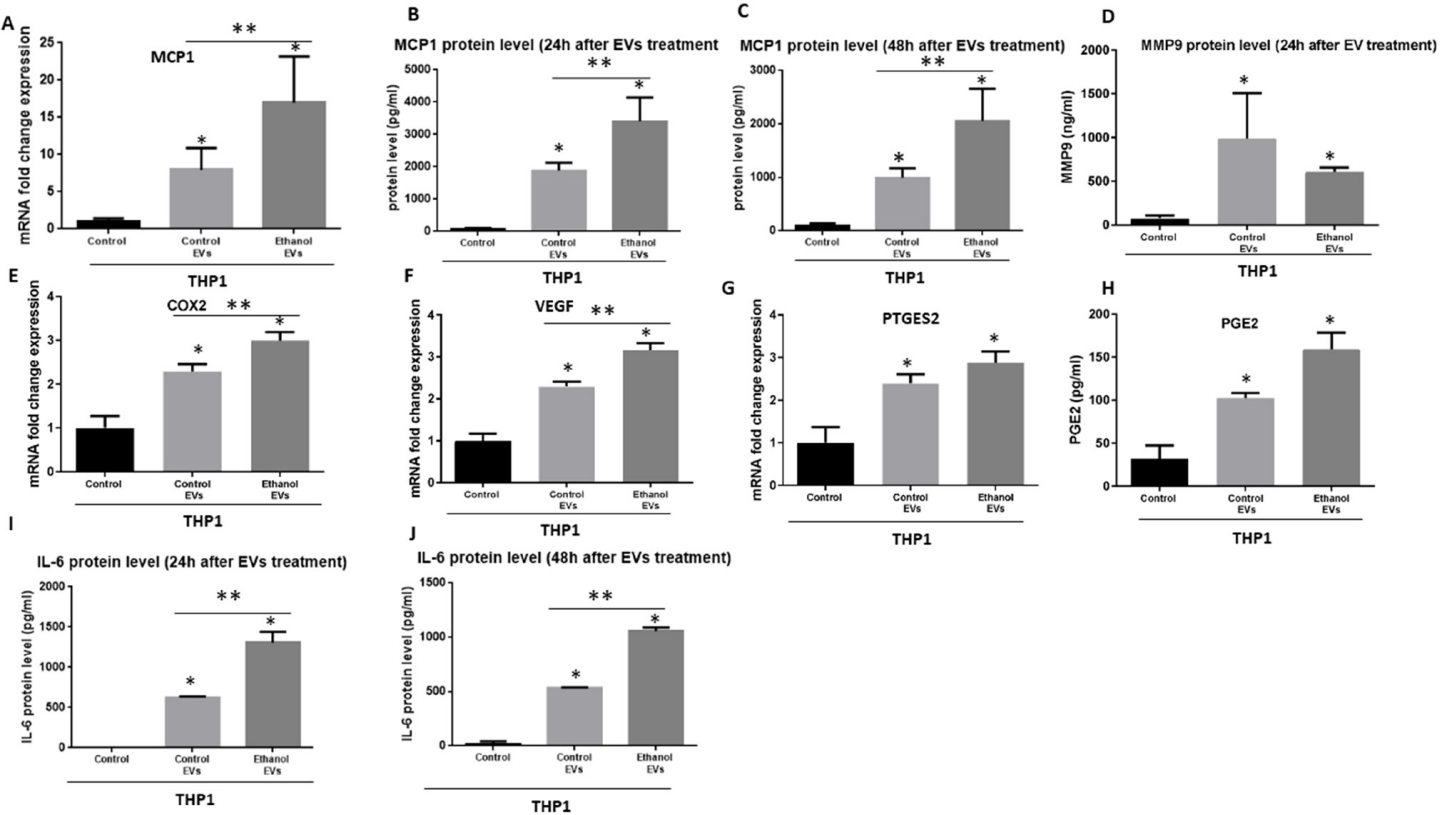
Immunomodulatory effects of EVs derived from OSCC cells (CAL27) on human THP1 monocytes (**A**) MCP1 mRNA level was determined by qPCR after 12 h co-culture of OSCC EVs with THP1 monocytes. (**B**, **C**) MCP1 protein levels were determined by ELISA 24 h and 48 h after coculture with EVs derived from OSCC cells. (**D**) MMP-9 protein levels were identified in the supernatant of THP1 cells 24 h after challenge with OSCC EVs. (**E**) mRNA levels of COX2 was quantified by qPCR. (**F**) VEGF mRNA level was measured by qPCR (**G**). mRNA level of PGE2 was quantified by qPCR. For real time PCR, 18s was used as endogenous normalizer. (**H**) Protein levels of PEG2 was determined by ELISA. (**I**, **J**) Protein levels of IL-6 were quantified in the supernatant of THP1 cells by ELISA in different conditions. (^*^indicates *p* < 0.05 *versus* control; ^**^indicates *p* < 0.05 between marked groups).

It has been evidenced that EVs have the ability to modify tumor microenvironment and circulating immune cells [21, 22]. Our results suggest activation of NF-κB by EVs in recipient THP1 monocytes, therefore, we postulated that EVs derived from OSCC could modulate tumor-associated monocyte/macrophages activity and may influence cancer biology. We further characterized the effect of EVs derived from CAL 27 cells on cytokine and chemokine production profile of THP1 cells. Monocyte chemoattractant protein-1 (MCP-1) is one of the derivers of tumor progression which molds the tumor microenvironment and promote the pro-oncogenic environment [41, 42]. 12 hours after co-culture of EVs derived from CAL27 cells the MCP1 mRNA level was increased significantly in recipient THP1 monocytes (Figure 6A). Consistently, MCP1 protein level was significantly increased 24 h and 48 h after co-culture of EVs with THP1 monocytes (Figure 6B, 6C). EVs derived from alcohol treated CAL27 cells further augmented the levels of MCP1 (mRNA and protein) in recipient THP1 monocytes (Figure 6A–6C). Level of pro-tumorigenic and pro-metastasis MMP9 protein, COX2 mRNA levels, PEG2 mRNA levels, and VEGF mRNA levels were increased significantly 24 h after co-culture of EVs with THP1 cells (Figure 6D–6G). We also found increased protein levels of PGE2 24 h after coculture with EVs (Figure 6H). Moreover, IL-6 protein levels were increased 24 h and 48 h after co-culture with EVs (Figure 6I, 6J). Enhanced levels in COX2 (Figure 6E), VGEF (Figure 6F), and IL-6 (Figure 6I, 6J) were found in THP1 monocytes after co-culture with EVs derived from alcohol treated CAL27 cells. These data suggest first EVs derived from CAL cells alone can reprogram THP monocytes via modulating NF-kB pathway and second, alcohol treatment further enhances these effects. Collectively, these data provide the evidence of in part modulating immune function to establish a pro-inflammatory milieu by miR-21.

**Figure 7:**
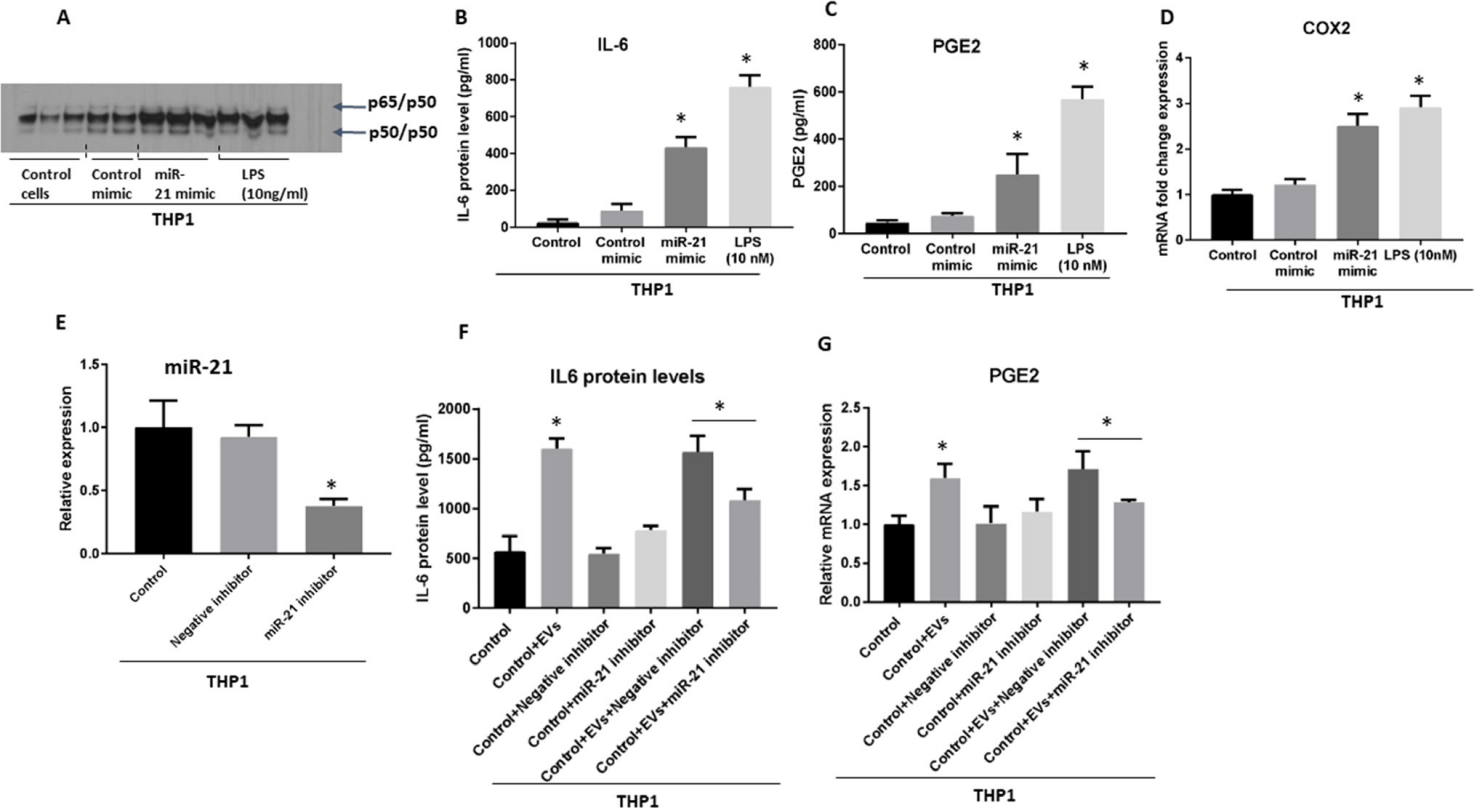
Gain- and loss- of function of miR-21 reveals its mechanistic role in the induction of pro-inflammatory phenotype in monocytes (**A**) miR-21 (at concentration of 150 pmol) or control mimic were electroporated into the human THP1 monocytes. THP1 cells were treated or not with 10 ng/ml LPS for 6 h, and 10 μg of whole cell lysate were subjected to EMSA to evaluate NF-κB activation. (**B**) Level of IL-6 was quantified by ELISA in the supernatant of THP1 cells 24 h after the introduction of miR-21 mimic. (**C**) Level of PGE2 was quantified by ELISA in the supernatant of THP1 cells 24 h after the introduction of miR-21 mimic. (**D**) COX2 mRNA was quantified using qPCR from cells 24 h after the introduction of miR-21 mimic. (**E**) miR-21 inhibitor (at concentration of 200 pmol) or control inhibitor were electroporated into the human THP1 monocytes and efficacy of inhibition was measured at 24 h. (**F**) IL-6 protein levels were quantified by ELISA in the supernatant of THP1 cells 72 h after the introduction of miR-21 inhibitor. (**G**) Level of PEG2 was quantified by qPCR in the THP1 cells 48 h after the introduction of miR-21 inhibitor. (^*^indicates *p* < 0.05 *versus* control; ^**^indicates *p* < 0.05 between marked groups).

To further delineate that the observed NF-κB activation was atleast dependent on miR-21 transfer, we transfected THP monocytes with miR-21 or control mimic and found the similar observation what was observed with EV treatment (Figure 7A). Importantly, miR-21 overexpression alone caused the NF-κB activation (Figure 7A). Consistently, protein levels of IL-6, and PGE2, and COX2 mRNA were increased 24 hours after introduction of miR-21, consistent with our findings of the role of miRNA in the activation of pro-inflammatory phenotype in monocytes (Figure 7B–7D). To further test the mechanistic role of miRNA-21 in the induction of pro-inflammatory phenotype loss of function experiment was performed by electroporation of miR-21 inhibitor (Figure 7E). After challenging THP1 monocytes with EVs derived from OSCC cells we found significant attenuated level of IL-6 protein (Figure 7F) and PGE2 mRNA (Figure 7G) in cells after the introduction of miR-21 inhibitor compared to miR-21 control inhibitor treated cells. These results confirm the mechanistic role of miRNA-21 in part in the induction of pro-inflammatory milieu in monocytes.

These results indicate the fact that high content of miR-21 in EVs, at least partially, explain the activation of NF-κB and observed pro-inflammatory profile. The amount of miRNA mimic that was incorporated into the cells was comparable to the amount of miR-21 presents in EVs isolated from 100 μl of the blood of patients with OSCC.

## DISCUSSION

Extracellular vesicles (EVs) are historically considered to be membrane-derived cellular debris with no biological or clinical significance produced during cell death. However, the evidence is amassing that EVs can exert multiple physiological and pathological functions as an essential mediator of intercellular communications [7, 20, 43]. The term ‘EVs’ collectively refers to a heterogeneous vesicular population spanning 50 to 10,000 nm in size. Distinct subpopulations include exosomes, microvesicles/microparticles, and apoptotic bodies, although the terms not associated with cell death have been used interchangeably in the past [44]. These EVs are secreted from almost all cell types into the aqueous extracellular microenvironment and represent a snapshot of the cell status at the time of release, as defined by their components [7, 8]. Beyond size, which is itself inadequate [45], morphological characteristics such as density, mode of biogenesis, and molecular markers such as CD63, CD81 and AnnexinV are used to classify EVs [44].

Recently, EVs have emerged as interesting research investigations in both the early detection of malignant tumors and signaling cascade between cells [18]. EVs, due to their natural ability to transfer biomacromolecules, have also been used as safe vehicles for the delivery of targeted drugs, as well as miRNAs, siRNAs, and miRNA inhibitors [7, 46]. Role of EVs was underlined in various level of the immune escape, drug resistance, formation of premetastatic niche and angiogenesis of tumors [46–48]. However, little is known about the role of EVs in cellular crosstalk in head and neck cancer and their possible mechanistic roles. It has been shown that a great proportion of miRNAs was associated with EVs and therefore are remarkably stable, and protected from endogenous RNase activity [18, 44, 49].

In this study, we showed that EVs are increased in the circulation of patients with OSCC and those EVs carry a set of oncogenic miRNAs. We isolated and characterized the EVs derived from OSCC cells and demonstrated that these are transferable to monocytes. We also characterized the miRNA cargo of OSCC-derived EVs and parental cells in the unstimulated state and stimulated with alcohol, a known OSCC risk factor. A set of miRNA was enriched explicitly in EVs, and functional network analysis showed that the novel EV associated miRNA signature of miR-22-3p, miR-130a-3p, miR-20a-5p, miR-21-5p, miR-195-5p, miR27a-3p, miR-19a-3p regulates different cancer- and innate immunity associated pathways. miR-21 was among the highly expressed miRNAs in both circulations of patients with OSCC and OSCC cells, therefore we determined the biological role of this miRNA in current study. Moreover, the level of miR-21 was increased in the EVs isolated from OSCC cells that were challenged with alcohol. We showed that EVs derived from cancer cells could be taken up by monocyte/macrophages and activate the NF-κB pathway through horizontal transfer of miR-21.

Endogenous RNAs acting as damage-associated molecular patterns (DAMPs) for pattern recognition receptors (PRRs) and may induce such signals [50]. Although these RNAs must remain unrecognized under non-pathological conditions, unshielded RNA packaged into the EVs is capable of inducing inflammatory responses in cancer cells [50]. Consistent with this concept we showed that EVs and EV-associated miR-21 could activate NF-κB pathway and induce inflammation in monocytes. Activation of NF-κB is part of the immune defense, which targets and eliminates transformed cells. This seems to be particularly true for acute inflammatory processes, where full activation of NF-κB is accompanied by high activity of cytotoxic immune cells against cancer cells. This immune defense against cancer cells, however, is normally not tight enough to eliminate all the aberrant cells, resulting in a shift to an equilibrium phase, which is often followed by an “escape” phase of the cancer cells, in which they outperform the immune system [51].

In this study, we found the horizontal transfer of miR-21 from OSCC cells to human monocytes. The *de novo* increased expression of miR-21 has been ruled out since levels of pri-miRNA-21 were unchanged in the cells challenged with EVs compared to the controls. However, in the later time points (18 h) we observed a significant increase in the pri-miRNA-21 expression indicating the downstream *de novo* expression of this miRNA after induction of NF-κB activation by tumor-derived EVs. Consistent with our findings other studies also reported the *de novo* expression of miR-21 as a downstream of NF-κB activation in cells with monocytic lineage [52]. In another report, miR-21 mimics induced more than a 2-fold induction of pro-tumorigenic IL-6 mRNA levels in THP1 cells compared with the control mimic [53], which is consistence with the effect of miR-21 that we observed in THP1 monocytes. Our data suggest that EVs horizontally transfer miR-21 and further activate surrounding immune cells to produce more pro-tumorigenic cytokines.

The inflammatory response and NF-κB activation have been reported to be associated with the formation of the pre-metastatic niche and cancer progression [54]. Specifically, activation of NF-κB usually leads to overexpression of anti-apoptotic genes thereby promoting cell survival mechanism to survive the cellular stress that propagated by the inflammatory response. Role of NF-κB pathway has been signified in controlling epithelial to mesenchymal transition as well as metastasis [55]. Additionally, NF-κB activation produces cytokines that regulate the immune response (such as IL-6, TNFα, and IL-8), which orchestrates the presence of leukocytes to the sites of inflammation [54, 56]. The mechanistic role of NF-κB in promoting metastasis involves up-regulation of matrix metalloproteinases (MMPs) which provide the escape environment for tumor evasion [57]. In the present research, we demonstrated that EVs derived from cancer cells could be taken up by monocytes and activate NF-κB in the recipient cells. The activation of NF-κB increased the pro-inflammatory cytokines such as IL-6, PGE2, and MCP1.

Monocytes are critical for the formation of tumor microenvironment and the initiation of tumor angiogenesis because they adhere to and invade endothelium activated by the increased shear stress that results from large pressure differences between perfused areas. MCP1 is one of the pivotal chemokines that regulate migration and infiltration of monocyte/macrophages and has been suggested as one of the major players of monocyte migration in the tumor microenvironment. The secretion of MCP1 by tumor-infiltrating immune cells, malignant cells, and other stromal cells suggests that MCP1 mainly supports tumor progression MCP1 attracts monocytes and also promote macrophage infiltration and macrophage-mediated angiogenesis [58, 59]. Expression of both MCP1 and VEGF have been positively associated with the presence of more tumor-associated macrophages and angiogenesis [60]. Consistently, we found increased levels of VEGF and MCP1 expression in monocytes after co-culture with tumor-derived EVs. We also found an increased production of PGE2 in human THP1 monocytes after co-culture with EVs. PGE2 is generally considered to possess strong pro-tumorogenic activity [61]. In a gain of function experiment, we delivered miR-21 in the comparable amount found in the circulation of patients with OSCC into the THP1 cells and found a comparable pro-inflammatory response in the monocytes. In the loss of function experimental setting, the introduction of miR-21 inhibitor to the THP1 cells attenuated the pro-inflammatory profile seen after challenge with OSCC-derived EVs. These results indicated that EV-medicated pro-inflammatory profiling of THP1 cells could at least partially be explained by the horizontal transfer of miR-21 via EVs.

In the present study, we showed that oncomiR such as miR-21 and miR-27 family are present with high abundance in the circulation of patients with oral cancer. EVs isolated from the circulation of OSCC patients and an OSCC cell line showed comparable miRNA profile, indicating the tumor origin of EVs in the circulation of patients with OSCC. In a series of experiments, we showed that EVs were taken up shortly by human monocytes after co-culture. Uptake of EVs derived from OSCC cells by monocytes caused activation of the inflammatory pathways, specifically NF-κB activation, and EVs conferred pro-tumorigenic properties and activated pro-tumor effectors in the recipient monocytes. These findings have possible implications for developing OSCC biomarkers and understanding and targeting the molecular and vesicular effectors in the tumor microenvironment.

## MATERIALS AND METHODS

### Cell culture and EV isolation

CAL27 cells were purchased from ATCC and were cultured in Dulbecco's modified medium (DMEM) plus 10% exosome-depleted FBS (Exo-FBS™) (Mountain View, CA, USA), and 1% penicillin/streptomycin (Gibco^®^, NY, USA). THP1 monocytes were cultured in RPMI media containing 10% exosome depleted FBS and 1% penicillin/streptomycin at 37°C in a 5% CO_2_ atmosphere. For evaluation of effects of ethanol administration, 25 mM, 50 mM, and 100 mM of ethanol were added to the cells for various time points (24 h, 48 h, and 72 h). At desired time points, conditioned media was collected, and EVs were isolated or quantified using a Nanoparticle Tracking Analysis (NTA) system as described previously [62].

For EV isolation from CAL27 cells, supernatants were centrifuged at 1500 g for 8 minutes to remove cells. Afterward, centrifugation at 10000 g for 20 minutes was done to deplete residual cellular debris. The supernatant was used to isolate EVs based on a polymer precipitation method with ExoQuick-TC™ (System Biosciences, USA). The manufacturer guidelines were used to isolate EVs. EVs were re-suspended in PBS for further analysis or functional experiments. For co-culture experiments, EVs isolated from OSCC cell line (CAL27) were added to THP1 cells for 2 h in the concentration of 85 μg/ml. This concentration was comparable to EVs concentration in the human circulation of human subjects with OSCC. After 2 h of co-culture of EVs with THP1 cells, THP1 cells were sedimented by centrifugation at 1000 RPM. 1000 RPM is capable of sedimenting cells, based on the density of EVs they are unlikely to co-participate with the THP1 cells. In fact, our quality control analysis showed EVs to be in the supernatant fraction. For ruling out any possible co-precipitation of EVs with cell pellet, cells were washed two times with PBS, centrifuged and harvested. 10 nM LPS was added 6 h before the readouts in the group, which involved LPS challenge.

### Isolation of EVs from plasma

For EV isolation from the plasma of human subjects (*n* = 34), EVs were isolated from 100 μl of plasma with ExoQuick reagent (System Biosciences, USA) according to the recommended protocol by the manufacturer. Patient samples were obtained from the University of Massachusetts Medical School Conquering Diseases Biorepository that were collected based on an institutional review board guidelines.

### Electrophoretic mobility shift assay (EMSA)

NF-κB activation in THP1 monocytes was evaluated after 6 h co-culture of EVs or 10 ng/ml LPS with the cells. Whole cell lysate was isolated, and 10μg of protein from THP1 monocytes was labeled with NF-κB probe consensus ds NF-κB oligonucleotide (5′-AGTTGAG GGGACTTTCGC-3′) End labeling was performed using T4 polynucleotide kinase in the presence of [γ-32P] ATP (Perkin Elmer, USA). Labeled oligonucleotides were purified with polyacrylamide copolymer column (Bio-Rad, USA). 10 μg from each sample was added to a reaction mixture containing 20 mM HEPES (pH 7.9), 50 mM KCl, 0.1 mM EDTA, 1 mM DTT, 5% glycerol, 200 μg/ml BSA, 2 μg of polydeoxyinosinic-polydeoxycytidylic acid, and γ-32P-labeled NF-κB oligonucleotide and incubated at room temperature for 30 mins. All reactions were run on a 6% polyacrylamide gel, and dried gel was exposed to X-ray film and kept at −80°C. For cold competition control, a 20-fold excess of the specific unlabeled double-stranded probe was added to the reaction mixture before adding the labeled oligonucleotide.

### Characterization of EVs

EVs were characterized by Nanoparticle Tracking Analysis (NTA), scanning electron microscopy (TEM), and Western blot regarding concentration, size, morphology, and surface marker respectively.

### Nanoparticle tracking analysis (NTA)

The concentration and diameter of EVs derived from plasma of human subjects and culture supernatant were identified by a NanoSight NS300 system (NanoSight, Amesbury, UK) equipped with fast video capture and Nanoparticle Tracking Analysis (NTA) software. Before each measurement, the instrument was calibrated with 100 nm polystyrene beads (Thermo Scientific, California, USA) as recommended by the manufacturer. The samples were recorded for 90s at controlled room temperature. NTA software was utilized to quantify the concentration of the particles (particles/ml) and size distribution (in nanometer). Measurements were done in triplicates.

### Western blotting

After isolation of EVs, the presence of EV markers, CD63 was assessed with western blot. Briefly, RIPA buffer was (Thermo scientific, USA) added to the EVs; EV proteins were extracted and run on 10% SDS-PAGE gels. Proteins were transferred to nitrocellulose membrane and were blocked for 1 h in TBS containing 5% non-fat dry milk and 0.1% Tween-20. The blots were incubated overnight with primary CD63 antibody (Abcam, Cambridge, USA) at 4°C. The blots were washed 3 times with TBST and then incubated for 1 hour with horseradish peroxidase-conjugated secondary goat anti-mouse IgG-HRP antibody (Abcam, Cambridge, USA) (dilution 1:10,000). Chemiluminescent Protein bands were detected using a Clarity™ Western ECL substrate kit (BioRad, USA) with an exposure time of 30s and analyzed with a Fujifilm LAS-4000 luminescent image analyzer.

### Confocal microscopy

EVs were collected from the supernatant of CAL 27 cells and were labeled with PKH67 green fluorescent cell linker kit (Sigma-Aldrich, USA). Co-culture of EVs with THP1 cells was done for 2 h, and then cells were washed out thoroughly with PBS 3 times to remove EVs. After 2 h, an uptake of labeled exosomes by recipient THP1 cells were visualized by a Leica TCS SP5 II laser scanning confocal microscope equipped with a 63X phase objective. Nomarski interference microscope was used to contrast the plasma membrane and nuclei were stained with DAPI (blue). Confocal stacked images (0.2 μm stack step, 1 μm range) were acquired and the Leica Application Suite (LAS) which integrates Leica automated microscopes images was used to construct 3D projections of image stacks.

### RNA isolation and small RNA profile

Both cells and exosomes were lysed in QIAzol Lysis reagent (Qiagen, Maryland, USA) and total RNA was isolated using miRNAeasy kit (Qiagen, Maryland, USA.) 100 μL of EVs suspension from plasma of patients, control subjects and, from supernatant of ethanol-treated cells, or normal cells were mixed with 400 μL QIAzol lysis buffer, and the mixture was processed according to the standard recommended protocol to extract the RNA. RNA was eluted with 30 μL of RNase-free water. The quantity and quality of the RNA were determined by NanoDrop 1000 (260/280 and 260/230 ratios) for cells. Aliquots of ~10 ng were submitted to capillary electrophoresis on a Bioanalyzer small RNA chip (Agilent). RNase protection assay was performed as previously described to confirm the intraluminal topology of EV associated extracellular RNA (25).

### Quantitative polymerase chain reaction (qPCR)

First, cDNA was transcribed from 500 ng of total RNA utilizing iScript™ cDNA synthesis kit (Bio-Rad) in a final volume of 20 μl. SYBR-Green-based real-time quantitative PCR (qPCR) was performed using the AB7300 Thermocycler, and the Bio-Rad CFX96 Real-time PCR Detection system was used for qPCR. The primer sequences were as follows: human GAPDH, forward, 5′-AGGGCTGCTTTTAACTCTGGT-3′; and reverse, 5′-CCCCACTTGATTTTGGAGGGA-3′; 18 S, forward, 5′-GACCTCATCCCA CCTCTCAG-3′, and reverse, 5′-CCATCCAATCGGTAGTAGCG-3′; human VEGF, forward, 5′-TTCATGGATGTCTATCAGCG-3′, and reverse, 5′-CATCTCTCCTATGTGCTGGC-3′; human MCP1, forward, 5′- CCCCAGTCACCTGCTGTT AT -3′, and reverse, 5′- TGGAATCCTGAACCCACTTC-3′; human COX2, forward, 5′- CCTTCCTCCTGTGCCT GATG-3′, and reverse, 5′-ACAATCTCATTTGAAT CAGGAAGCT3′; human PTGES2, forward, 5′- CTT CCTTTTCCTGGGCTTCG-3′, and reverse, 5′- GAAGAC CAGGAAGTGCATCCA-3′. 18s or GAPDH were used as an endogenous normalizer, and relative expression levels were calculated using the ΔΔCt method.

### TaqMan MicroRNA assays

TaqMan^®^ miRNA Assays (Applied Biosystems, Foster City, USA) was utilized for quantification of miR-21, miR-27a, miR-155, miR-27b expression according to the manufacturer's protocol. Reverse transcription step was as the following: 30 min, 16°C; 30 min, 42°C; and 5 min 85°C. miRNA reverse transcription was done with the mixture of TaqMan stem-loop primer, 10 ng RNA, TaqMan primers and kit according to the manufacturer's recommendation. qPCR was using TaqMan Universal PCR Master Mix and recommended protocol by the manufacturer. RNU48 was used to normalize the Ct values between the samples. In experiments involving miRNA analysis of EVs and patients' plasma, the same volume of starting material was used and synthetic C. elegans (cel)-miR-39 was spiked during the total RNA isolation process and used to normalize the qPCR data. TaqMan^®^ Pri-miRNA for miR-21 Assay was performed using FAM dye-labeled TaqMan with GAPDH as an internal control. Experiments were done in triplicate. Levels of miRNAs were normalized, and the relative expression levels of each miRNA were presented and analyzed by 2–ΔΔCt as described by Livac and Schmittgen (26).

### Transfections via electroporation

For the direct introduction of miR-21 mimic (150 pmol) or miR-21 inhibitor (200 pmol) (Ambion, USA), THP1 cells were transfected by electroporation as follows using our previously established method (7). Briefly, 2 × 10^5^ cells were re-suspended in 150 μl complete RPMI media and 150 μl Gene Pulser^®^ electroporation buffer for 10 min on ice before being electroporated. Electroporation was done at 300 kV and 1500 μF. Following electroporation, cells were kept on ice for 10 min then cultured in media for 24 hrs. Similar concentrations of miR control mimics and negative inhibitor were introduced to the THP1 cells using similar approach and conditions.

### Enzyme-linked immunosorbent assay (ELISA)

Levels of TNFα (R&D Systems, Minneapolis), MCP1 (BioLegend, San Diego), prostaglandin E2 (PGE2) ELISA Kit (Abcam, Cambridge), IL-6 (R&D Systems, Minneapolis), MMP9 (Abcam) and IL-1β (R&D Systems, Inc., Minneapolis) were measured from cell free supernatants following manufacturer's recommendations after construction of standard curve and measured using an ELISA reader. Results were expressed as mean ± SD. The statistical significance was determined between two groups with the student's *t*-test after one-way analysis of variance.

### Microarray analysis and pathway enrichment analysis

The Firefly miRNA multiplex assay (Firefly™ bioworks, Cambridge, USA) was used to profile EV-associated miRNA isolated from CAL27 cells and cells in different conditions (non-challenged and challenged with 25 mM ethanol) for a targeted list of 68 miRNAs in the oncogenic panel. Total RNA (12 μl) was used to run the Firefly standard protocol and the abundance of RNA probes analyzed with a Millipore Guava 8HT flow cytometer (EMD Millipore). Analysis and visualization of data were performed using a Firefly™ Analysis Workbench. Intensities were transformed to log ratio for each probe. Negative control values for each probe was subtracted from each sample and normalization was done based on the geometric mean of most stable reference genes based on the software built in the algorithm. Signal log ratios were computed by using a *t*-test and false discovery rate was controlled at 5% using the Benjamini-Hochberg procedure. The identified miRNAs were clustered in by K mean clustering method and heat-map was constructed. The miRNAs that were specifically enriched in EVs were subjects to functional network analysis through miRNet which combine the target interaction data from 11 well-annotated miRNA datasets including PhenomiR, SM2miR, TarBase, HMDD, PharmacomiR and miR2Disease (27). Network visualization and functional enrichment analysis were performed based on Reactome, Gene Ontology, and KEGG to identify dysregulated pathways associated with enriched miRNAs in EVs by a hypergeometric test as well as small molecules interactions (27).

### Statistical analysis

After determination of data distribution, one-way analyses of variance (ANOVA) or Kruskal-Wallis nonparametric test were used to compare different groups. Student's *t*-test or Mann–Whitney *U* test was for pair-wise group comparisons. Data are presented as a mean ± standard error of the mean (SEM) or standard deviation (SD) and *P* values of less than 0.05 were identified as statistically significant.

## SUPPLEMENTARY MATERIALS FIGURES AND TABLES






