# Supplementary material for: Extracellular vesicles in oral squamous carcinoma carry oncogenic miRNA profile and reprogram monocytes via NF-κB pathway
Source: Oncotarget. 2018 Oct 5;9(78):34838–54. doi: 10.18632/oncotarget.26208 (PMC6205181; doi:10.18632/oncotarget.26208)
Supplement: Supplementary file 1 [file oncotarget-09-34838-s001.pdf]

Extracellular vesicles in oral squamous carcinoma carry oncogenic miRNA profile and reprogram monocytes via NF-κB pathway

SUPPLEMENTARY MATERIALS

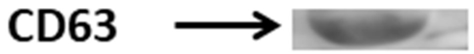

Supplementary Figure 1: Expression of CD63 in Evs derived from CAL27 cells.

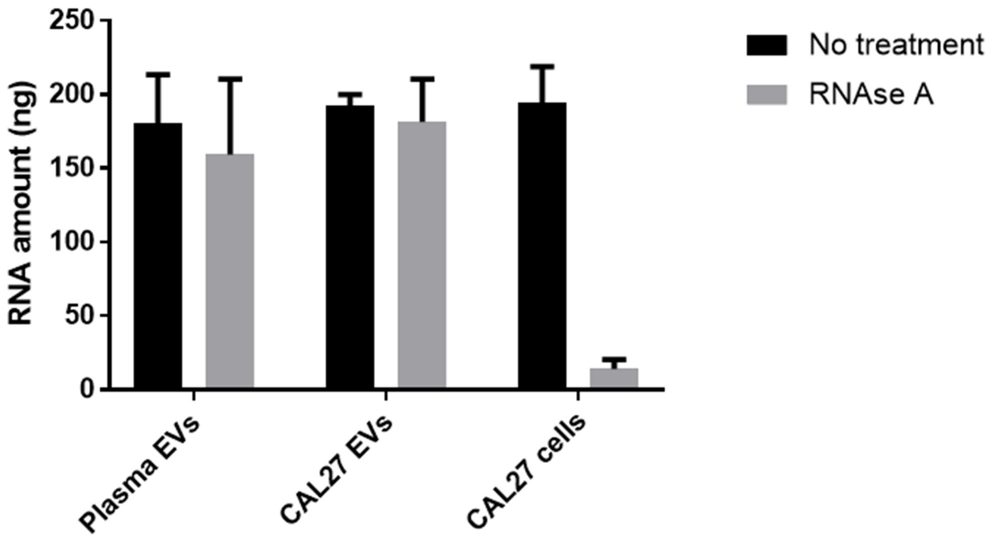

Supplementary Figure 2: Total RNA yield in plasma EVs, CAL27 Evs and CAL27 cells with and without RNase treatment.

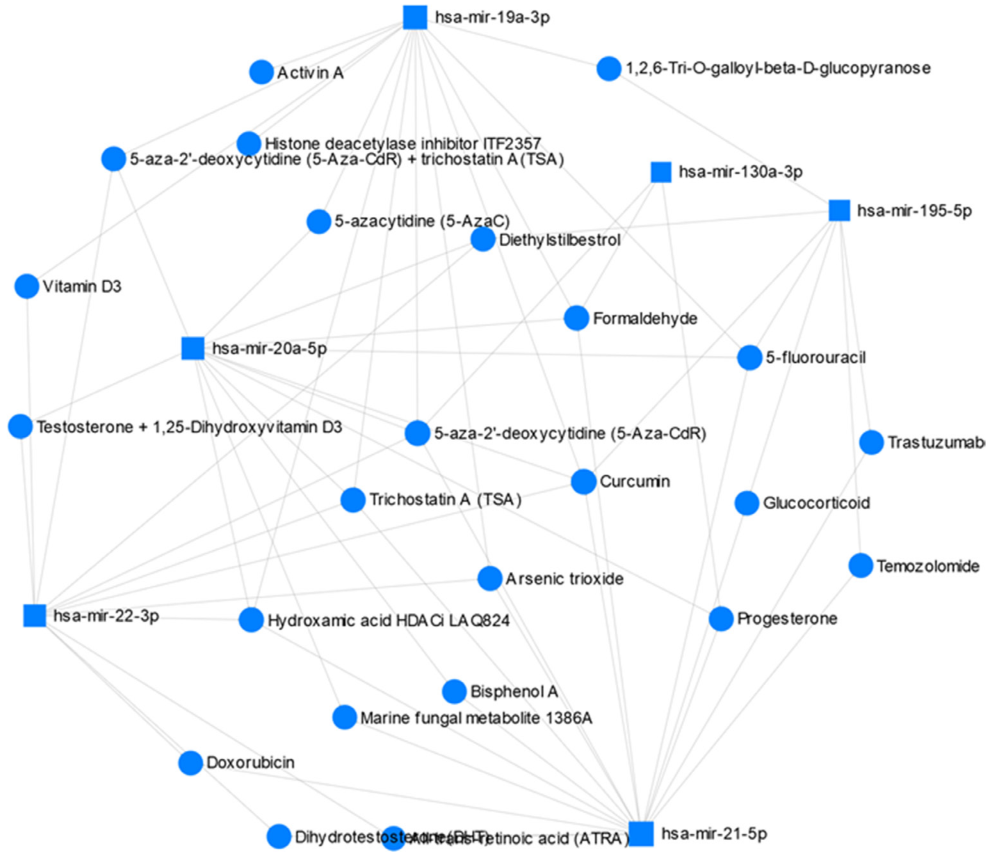

Supplementary Figure 3: Small molecules targeted by EV miRNAs dervied from OSCC cancer cells.

Supplementary Table 1: Demographics and other characteristics in the study samples

	Total (*n* = 34)	OSCC (*n* = 24)	Controls (*n* = 10)
Gender (male/female)	31/49	14/24	6/10
Age; mean (SD)	63.61 (13.63)	65.73 (13.92)	62.36 (12.81)
Race			
Asian	6	3	3
Black/African American	3	3	0
White	24	17	7
Location of malignancies*; *n*			
Alveolar ridge	8	8	-
Tongue	13	13	-
Buccal mucosa	4	4	-
Palate	1	1	-
Tonsil	1	1	-

*Some patients contributed to more than 1 site.

Supplementary Table 2: KEGG pathway enrichment analysis of miRNA targets. See Supplementary_Table_2

Supplementary Table 3: Reactome pathway enrichment analysis of miRNA targets. See Supplementary_Table_3
